# Imaging Review of Subscapularis Tendon and Rotator Interval Pathology

**DOI:** 10.1155/2022/4009829

**Published:** 2022-01-11

**Authors:** Zohaib Y. Ahmad, Luis E. Diaz, Frank W. Roemer, Ajay Goud, Ali Guermazi

**Affiliations:** ^1^Department of Radiology, Boston Medical Center, Boston University School of Medicine, Boston, MA 02118, USA; ^2^Department of Radiology, VA Healthcare System, West Roxbury, MA 02132, USA; ^3^Department of Radiology, University of Erlangen-Nuremberg, Erlangen 91054, Germany

## Abstract

As the largest rotator cuff muscle, the subscapularis plays a major role in stabilizing the glenohumeral joint, in conjunction with surrounding rotator cuff structures. Injury to the subscapularis tendon can be isolated, but more commonly is seen in conjunction with supraspinatus tendon pathology. Injury can be associated with biceps pulley instability, superior labral anterior-posterior (SLAP) tears, humeral head subluxation, and anterosuperior and coracoid impingements. The involvement of the rotator interval can lead to what is called “the hidden lesion,” due to its difficulty to diagnose during arthroscopy. Understanding the anatomical relations of the subscapularis tendon with the rest of the rotator cuff and rotator interval, as well as common patterns of injury that involve the subscapularis tendon, can aid in proper diagnosis of these injuries leading to prompt surgical repair. This review describes the anatomy of the subscapularis muscle and tendon, and the magnetic resonance imaging (MRI) patterns of subscapularis tendon injury.

## 1. Introduction

The subscapularis muscle is the largest muscle of the rotator cuff. The rotator cuff, consisting of the supraspinatus, infraspinatus, teres minor, and subscapularis muscles, works in concert to facilitate movement and provide stability of the glenohumeral joint [1]. It is thought that the subscapularis prevents anterior dislocation of the humeral head from the glenoid fossa [1]. Functionally, the subscapularis works to internally rotate and adduct the humerus [2].

In this review, we will discuss subscapularis muscle and tendon anatomy, the magnetic resonance imaging (MRI) patterns of subscapularis tendon injury, and associated pathology, and argue that proper diagnosis may influence prompt surgical repair.

## 2. Anatomy and Function

**Figure 1:**
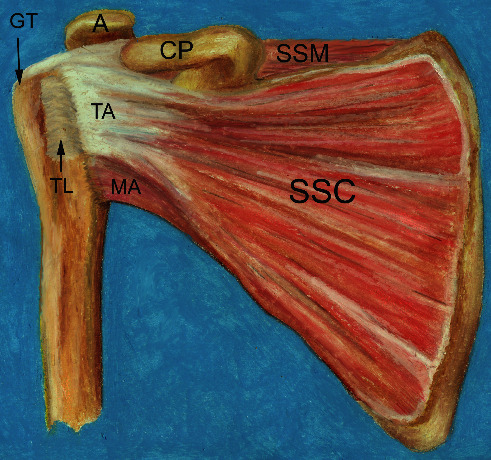
Anatomy of the subscapularis muscle. The subscapularis muscle (SSC) with its tendinous attachment (TA) and muscular attachment (MA) at the humerus. The transverse ligament (TL) covers the bicipital groove. The supraspinatus muscle (SSM) and tendon insert onto the greater tuberosity (GT). The acromion (A) and coracoid process (CP) are depicted (illustration courtesy of Virginia Diaz).

The subscapularis muscle (Figure 1), triangular in shape, arises as four to six collagen fiber bundles from the anterior surface of the scapula and posterior wall of the axilla to insert on the lesser tuberosity of the humerus, covering the anterior aspect of the humeral head [1, 3]. These collagen fibers merge with the supraspinatus fibers laterally to form the roof of the biceps tendon groove, which encompasses the long head of the biceps [1, 4, 5]. Inferior to the lesser tuberosity, the muscle inserts directly on the bone [6].

The muscle itself can be divided into two segments, the upper two-thirds and lower one-third, respectively. The muscle provides both passive stabilization and dynamic stabilization of the glenohumeral joint, buttressing the joint from a posteriorly directed force. The upper segment of the muscle has been shown to be more involved with internal rotation, while the lower segment can help in external rotation while the arm is abducted [7–9]. These portions of the muscle and their corresponding tendons are each innervated by the superior and inferior subscapularis nerves, a branch of the posterior cord of the brachial plexus [10].

**Figure 2:**
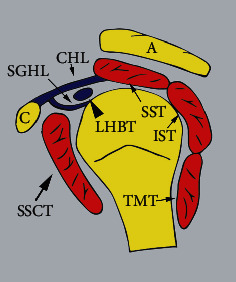
Anatomy of the rotator interval. Sagittal view of the humerus. The supraspinatus tendon (SST) and subscapularis tendon (SSCT) are traversing laterally superior and anterior to the humeral head. The long head of the biceps tendon (LHBT) is anterior to the SSCT after it originates from the superior glenoid. The coracohumeral ligament (CHL) originates from the coracoid process (C) as it extends between the SST and SSCT, abutting the superior glenohumeral ligament (SGHL). The acromion process (A) is also shown. IST = infraspinatus tendon; TMT = teres minor tendon (illustration by Zohaib Ahmad).

The superior aspect of the subscapularis tendon provides the inferior border of the rotator interval, a triangular area in the anterosuperior glenohumeral joint capsule that is reinforced by the superior glenohumeral ligament (SGHL) and the coracohumeral ligament (CHL). The anterior aspect of the supraspinatus tendon provides the superior border of the rotator interval, while the base of the coracoid process provides the lateral border [11–14]. The CHL traverses from the lateral aspect of the base of the coracoid, splitting into two bands: the lateral band crossing the biceps tendon to insert onto the greater tuberosity meshing with tendon fibers of the supraspinatus and the medial band inserting onto the lesser tuberosity and tendon fibers of the subscapularis (Figure 2). The SGHL arises at the supraglenoid tubercle to enmesh with the CHL and insert at the lesser tuberosity [12]. The SGHL also merges with the middle glenohumeral ligament to form a complex termed the medial sheath [11]. Both the CHL and the SGHL stabilize the long head of the biceps tendon (LHBT) within the bicipital groove along the anterior aspect of the humerus. The subscapularis tendon is intimately involved with these ligaments and with the stability of the LHBT, together forming the capsuloligamentous complex called the biceps pulley [11].

## 3. Epidemiology and Diagnosis

Rotator cuff tears are relatively common, usually occurring in patients over the age of 40, and are often degenerative in nature [15]. Tears of the subscapularis tendon usually occur in combination with tears of the supraspinatus tendon. Subscapularis tendon tears are relatively uncommon, tending to occur in a slightly younger population than those that suffer tears of the rest of the rotator cuff [1, 5, 9]. It has been described that 2–6% of rotator cuff tears involve the subscapularis [1, 5]. Isolated subscapularis tears are even more rare [16]. Furthermore, the mechanism of injury of these isolated tears is usually due to an acute trauma, commonly involving hyperextension or external rotation of the abducted arm [5, 9, 15, 16].

As described above, patients may present with anterior instability and dislocation; however, many patients do not present with any clinical symptoms at all [6, 7, 9]. Barlow and Everhart described that 40% of patients who had a torn subscapularis tendon upon arthroscopy had normal physical examination findings [17]. Recent studies have shown that many subscapularis tears can be easily missed and that the incidence of such tears may actually range from 30 to 50% [18, 19]. However, clinical diagnosis can be achieved through specific physical findings. An increase in passive external rotation and weakness of internal rotation will be noted. A pathological “lift-off test” involving extension and internal rotation of the arm, as developed by Gerber and Krushell, is specific for disruption of the subscapularis muscle [16, 20].

Even upon arthroscopy, subscapularis tendon tears can be difficult to diagnose, especially if they are partial-thickness tears or demonstrate no retraction. Certain arthroscopies have difficulty visualizing the subscapularis tendon attachment at the lesser tuberosity. While the subscapularis tendon is difficult to access and visualize upon arthroscopy, recent attention and improved arthroscopic techniques have increased the incidence of detection of such lesions [21–23].

## 4. Classification Schemes of Subscapularis Tendon Tears

No concrete MRI grading scheme has been developed for rotator cuff tears. Different classification systems have been suggested for MRI and arthroscopy. Lafosse suggested a grading scheme using arthroscopy, with five different grades based on the extent of the tear [23]. Pfirrman et al. used an MRI grading scheme, which involved normal tendon (Grade 0), tear involving less than quarter of the tendon (Grade 1), tear involving more than quarter of the tendon (Grade 2), and a full-thickness tear (Grade 3) [4]. Similar grading of tears is used during MRI to create cohesion between the imaging and surgical findings [4]. Many other grading schemes for the subscapularis muscle have emerged such as Lyons and Green, and Fox classifications, which are based on insertion site lesions [18, 24, 25]. A classification by Yoo and colleagues bases itself on the 3D footprint of the subscapularis muscle as a whole, given the many attachment sites and planes through which the subscapularis muscle travels, differentiating tendon and muscular tear [18].

## 5. The Importance of Diagnosis

As the subscapularis plays an important role in the glenohumeral and bicipital stability, failure to address and repair a torn subscapularis tendon can lead to persistent pain. Mall et al. found that concomitant repair of the subscapularis tendon alongside the supraspinatus tendon provided more pain relief than repairing the supraspinatus tendon alone [9]. Other studies support that repair of the subscapularis tendon alongside the supraspinatus tendon decreases stress on the adjacent supraspinatus repair [19].

Failure to repair a concomitant subscapularis tendon tear has been found to compromise the normal biomechanical function of the tendon as described above and potential compromise of an associated posterosuperior rotator cuff tear repair [25, 26]. Chronically torn tendons undergo muscular atrophy and fatty degeneration, which have been described as negative predictors for a successful surgical repair [4, 8, 19].

However, small tears are easily missed; Adams et al. described a sensitivity of 36% and specificity of 100% for diagnosing subscapularis tendon tears on MRI [27]. Associated signs such as biceps pulley lesions or impingement may strongly suggest that a subscapularis tear, isolated tears, unless complete and retracted on imaging, can be easily missed [19]. Many of these tears are close to the articular cephalad surface (greater than 90% per ward) [19]. Preoperative MRI has also been found to miss tears unless they extend to at least half of the subscapularis tendon in the cephalad or caudal direction [27]. It is possible that accurate 3D footprinting of the tendon as described by Yoo et al. may provide a more reliable way of diagnosing articular surface tears [18].

Adams et al. provided a systemic approach to diagnose a subscapularis tear on MRI, including axial cuts to evaluate subscapularis tears, axial cuts to evaluate subluxation of the biceps tendon, sagittal cuts to evaluate subscapularis atrophy, and sagittal images to evaluate subscapularis tears at the tendinous insertion onto the lesser tuberosity [28]. Accurate diagnosis of subscapularis tendon tears may provide value in preoperative preparation and accurate repair [21].

## 6. MRI Sequences

**Figure 3:**
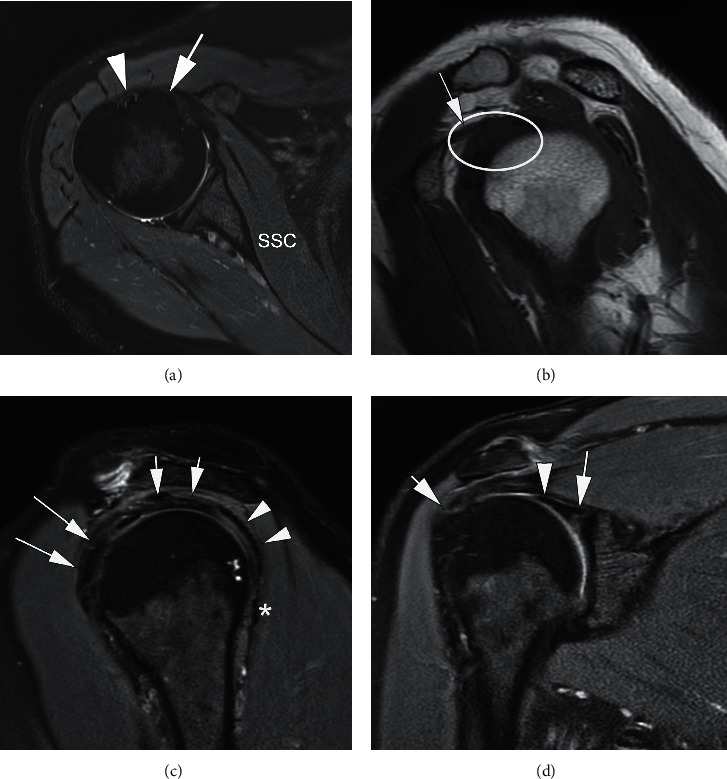
Normal MRI of the right shoulder. Axial T2-weighted fat-suppressed (FS) MRI (a) demonstrates the subscapularis muscle (SSC) traversing along the anterior aspect of the scapula to insert on the lesser tuberosity of the humerus (arrow). The long head of the biceps tendon (arrowhead) is correctly positioned in the bicipital groove at this level. Sagittal oblique T1-weighed MRI (b) provides a view of the rotator interval (ellipse) with visualization of the coracohumeral ligament (arrow). Sagittal oblique T2-weighed FS MRI (c) shows the lateral subscapularis (long arrows), supraspinatus (short arrows), infraspinatus (arrowheads), and teres minor (star) tendons close to their humeral attachment. Coronal T2-weighted FS MRI (d) shows the biceps anchor (arrow head) and the proximal horizontal portion of the long head of the biceps tendon close to its attachment at the glenoid (long arrow). *Note.* Tendinosis of the supraspinatus attachment is reflected as intratendinous hyperintensity (short arrow).

**Figure 4:**
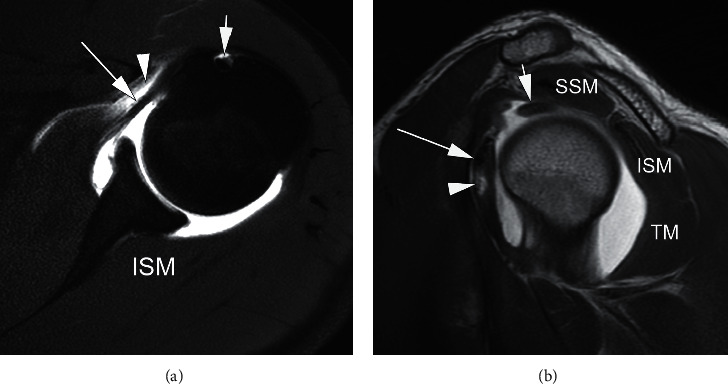
Normal MR arthrogram: axial T1-weighed FS (a) and sagittal T1-weighted (b) MRI show an intact subscapularis tendon (long arrow in a and b), long head of the biceps tendon (short arrow in a and b), supraspinatus muscle (SSM), infraspinatus muscle (ISM), and teres minor (TM). *Note.* In addition, there are some extra-articular contrast material deposition anterior to the subscapularis tendon (arrowhead in a) and minor contrast deposition within the musculotendinous junction of the subscapularis (arrowhead in b).

MRI and MR arthrography (MRA) of the shoulder joint usually include three planes including the axial, oblique coronal, and oblique sagittal planes. The subscapularis is best evaluated on axial and oblique sagittal planes [4]. Axial images allow visualization of the subscapularis tendon as it inserts into the lesser tuberosity and the long head of the biceps tendon as it travels in the bicipital groove (Figures 3 and 4) [15]. Furthermore, parasagittal images have shown a higher specificity for subscapularis tendon lesions when compared with axial imaging [4]. Overall, the sensitivity for the diagnosis of rotator cuff lesions on MRA can range from 71% to 100%, with full-thickness tears having a sensitivity of 100% and diagnosis of subscapularis tendon lesions having a sensitivity of 91% [4, 29]. MRI with or without intra-articular contrast has been found to have a sensitivity of 31–91% in diagnosing subscapularis tendon tears, quite different from the sensitivity of 80–100% in diagnosing supraspinatus tendon tears [25]. In a 2012 study by Foad and Wijdicks, MRA did not show an increased detection rate in diagnosing a subscapularis tendon tear [30].

## 7. Pathology of Subscapularis Tendon Tears on MRI

The pattern of subscapularis tendon tears has been described by Pfirrman et al. to include three types: isolated, involving the supraspinatus tendon, or involving the rotator interval [4].

### 7.1. Isolated Tears

**Figure 5:**
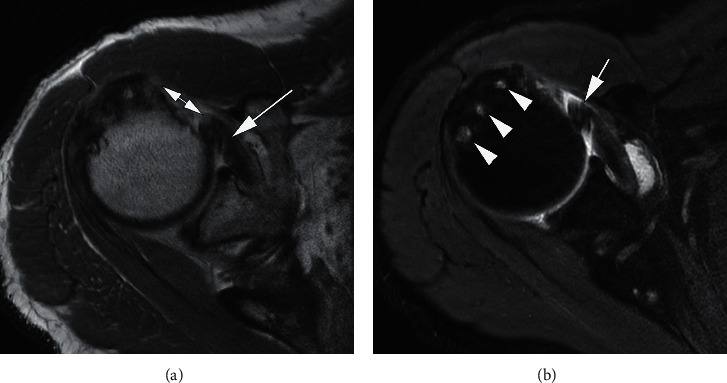
A 49-year-old man with right shoulder pain and inability to extend the right arm after a fall. Axial PD-weighed MRI (a) demonstrates a full-thickness subscapularis tendon tear (long arrow) with medial retraction (short double-headed arrow). Axial T2-weighed FS MRI (b) shows multiple subchondral cysts at the greater tuberosity (arrowheads), which is an associated finding with rotator cuff tears. In addition, there is a medial displacement of the long head of the biceps tendon (arrow).

Isolated tears of the subscapularis muscle have been described in the setting of anterior dislocation of the humeral head and recurrent anterior instability [4]. The most common site of tear is at the tendinous interface with the lesser tuberosity of the humerus; Ward et al. described that greater than 90% of subscapularis tendon tears occur along this articular surface [19] (Figure 5). The commonality of symptoms with other rotator cuff tears makes localization of subscapularis tears difficult [15]. On MRI, isolated complete subscapularis tendon tears will demonstrate poorly defined contours with fluid signal intensity (Figure 5). Less commonly, the tendon will show discontinuity and retraction of the tendon fibers [1, 15, 19].

Osseous changes, however, can unveil a subscapularis injury; lesser tuberosity cysts have been shown to be specific [2, 3] for tears alongside fatty atrophy of the muscle, a sign of chronic injury. The mechanism of these osseous changes is thought to be through either impingement of the lesser tuberosity or fluid intrusion through cortical infarctions [2].

### 7.2. Association with Tears of the Supraspinatus Tendon

**Figure 6:**
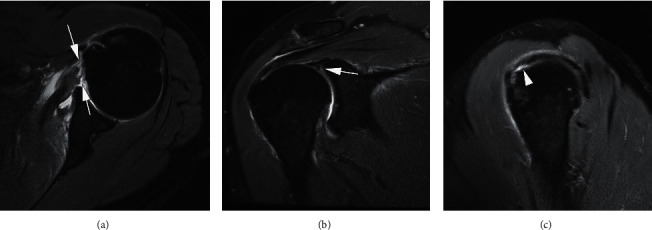
A 46-year-old man with left shoulder pain and limited range of motion after falling during football. Axial T2-weighted FS MRI (a) shows a full-thickness subscapularis tendon tear without relevant retraction from its attachment (arrows). In addition, there is a superior labral tear (arrow) seen on coronal T2-weighted FS MRI (b). Sagittal oblique T2-weighted FS MRI (c) demonstrates a small articular surface tear at the attachment site of the supraspinatus tendon (arrowhead).

More commonly, subscapularis tendon tears are associated with supraspinatus tendon tears (Figure 6), at the lesser tuberosity of the humerus [15]. These can appear as an extension of a large supraspinatus tear through the subscapularis tendon or as an anterosuperior rotator cuff lesion extending through structures such as the CHL and SGHL to involve the supraspinatus and subscapularis tendons [4].

### 7.3. Association with the Rotator Interval and the Biceps Pulley

**Figure 7:**
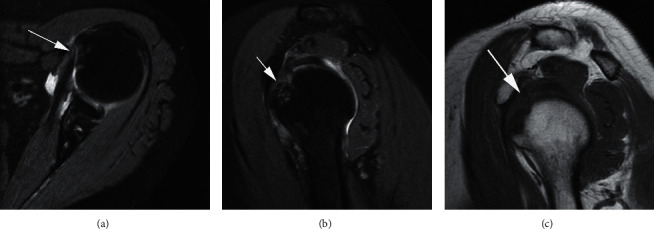
A 64-year-old woman with left shoulder pain after a fall. Axial (a) and sagittal oblique (b) T2-weighted FS MRI demonstrates a partial insertional tear of the subscapularis tendon (arrow). Sagittal oblique T1-weighted MRI (c) demonstrates loss of fat within the rotator interval (arrow).

**Figure 8:**
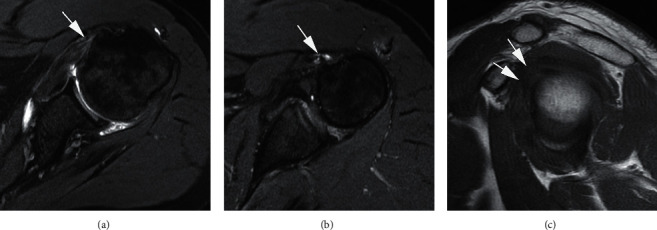
A 32-year-old man with left shoulder pain after dislocation. Axial T2-weighted FS MRI (a) and (b) show an articular surface partial tear of the subscapularis tendon (arrow in a) with tearing and medial dislocation of the long head of the biceps tendon (arrow in b). Sagittal oblique T1-weighted MRI (c) displays loss of normal fat within the rotator interval (arrows).

Injury to the rotator interval, of which the subscapularis tendon forms the inferior border, can produce what has been described as “hidden lesions” [12], due to the difficulty in diagnosis during arthroscopy. The injury will lead to biceps instability, with the potential involvement of the SGHL and CHL (Figures 7 and 8). Lee et al. described 27% of biceps pulley lesions involving the subscapularis and supraspinatus tendons and 47% of that involving the SGHL and CHL [13]. This association is further solidified in a grading system formulated by Bennet in 2001 [12, 13], which assesses the involvement of the subscapularis tendon, SGHL, CHL, and supraspinatus tendon [11]. Many tears of the anterior supraspinatus tendon and superior subscapularis tendon (anterosuperior rotator cuff) have been found to dissect into the CHL, which may also involve the medial sheath, otherwise known as the SGHL-MGHL complex [11] as demonstrated by the Bennet classification. Furthermore, full-thickness tears of the subscapularis tendon with humeral avulsion of SGHL-MGHL complex have been shown to medially sublux, presenting as a curved band of tissue obscuring the rotator interval upon arthroscopy, and dubbed the “comma sign” [22, 31].

The difficulty in diagnosis of rotator interval lesions lies in the variability of rotator interval appearance on MRI [14]. Failure to address these lesions upon arthroscopic repair produces persistent instability [11–14].

**Figure 9:**
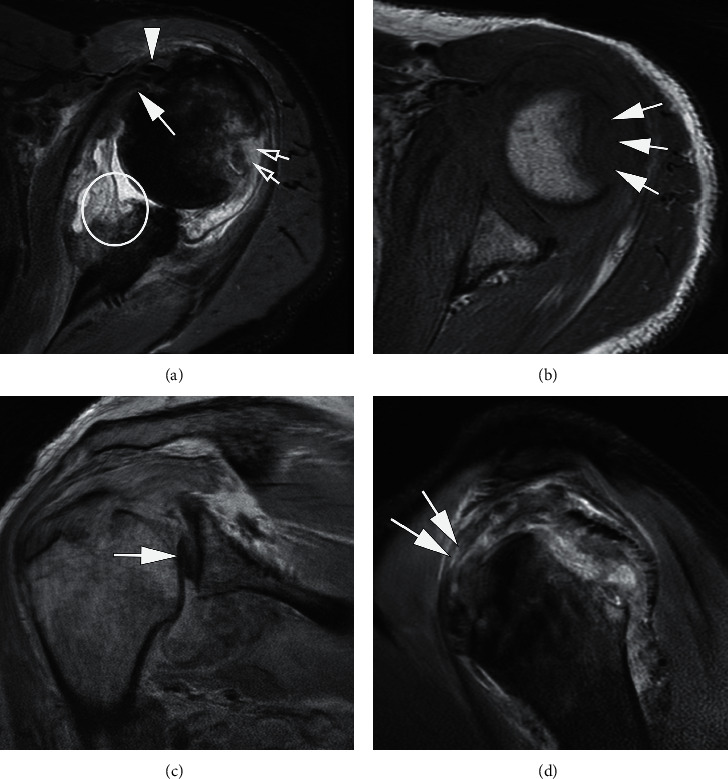
A 67-year-old man with persistent pain after a left shoulder dislocation. Axial T2-weighed FS MRI (a) shows a partial articular surface tear of the subscapularis tendon (large arrow) with mild medial displacement of the long head of the biceps tendon (arrowhead). Also evident are an osseous Bankart lesion (ellipse) and the corresponding Hill–Sachs deformity (two short arrows). The full extent of the Hill–Sachs depression is best appreciated in the axial PD-weighted MRI (b), consistent with recent anterior dislocation. Coronal PD-weighted MRI (c) demonstrates a superior labral tear with the displacement of a portion of labrum (arrow) into the glenohumeral joint space. Sagittal oblique T2-weighed FS MRI (d) shows an intrasubstance tear of the genu of the long head of the biceps (arrows).

Subscapularis tendon tears can also lead to biceps tendon instability and subsequent subluxation (Figures 5, 8, and 9). The pathogenesis for these lesions varies from degeneration, trauma, to sequelae of a rotator cuff injury [32]. Upon MRI, patients may show bicipital subluxation [15, 33] and anteromedial dislocation of the long head of the biceps tendon [1, 4, 32]. This medial dislocation has been said to be diagnostic for subscapularis tendon tears [4, 34]. One study demonstrated subluxation of the biceps tendon to be always associated with supraspinatus tendon tears and subscapularis tendon lesions [35], and Schaeffeler et al. posited that exclusion of biceps pulley lesions may avoid unnecessary diagnostic arthroscopy [34].

MRI-proven abnormalities of the superior aspect of the subscapularis tendon have shown a high sensitivity, specificity, positive predictive value, and negative predictive value with surgically proven biceps pulley lesion, even if the lesions of the biceps pulley were not evident by imaging [36]. In fact, Urita et al. demonstrated that those patients with subscapularis tendon tears on MRI were 6 times more likely to have a severe grade biceps tendon disorder found during surgery when compared to those without a subscapularis tendon tear [37].

### 7.4. Association with Impingement

Subscapularis tendon tears can also lead to anterosuperior impingement, a painful process from impingement of the deep tendinous surface of the subscapularis and the biceps pulley along the anterosuperior glenoid rim. This is elicited by horizontal adduction and internal rotation of the arm [13, 32]. These develop from, amongst other reasons, SGHL injuries with a partial subscapularis tendon tear [32]. Impingement has even been described as a marker for increased surgical failure [3].

On the other hand, subcoracoid impingement occurs when a part of the rotator cuff finds itself between the coracoid process and the head of the humerus, with a significant decrease in the average coracohumeral distance and the distance between the lesser tuberosity of the humerus and the coracoid process [3, 38] (Figure 9). A normal coracohumeral distance has been described as 11 mm, while Friedman et al. asserted that a patient will become symptomatic with a coracohumeral distance less than 5.5 mm [38]. However, there is debate in the literature on whether the severity of a subscapularis tendon tear is related to the measured subcoracoid interval [3]. Giaroli et al. described that the coracohumeral distance measurement may be only of use if the patient is symptomatic [39].

### 7.5. Association with SLAP Tears

Superior labral anterior-posterior (SLAP) tears may be seen in conjunction with subscapularis and proximal biceps tendon injuries (Figures 6, 8, and 9). Hawi et al. described that 47.2% of a biceps pulley lesion had a SLAP tear [40]. As the long head of the biceps tendon anchors upon the superior labrum, SLAP tears have a similar pathogenesis as biceps pulley lesions such as trauma including a fall on an outstretched hand or degeneration [13, 33]. It is described that the original dislocation event alters the biomechanical forces of the glenohumeral joint, damaging the anterior glenoid cartilage and labrum; recurrent shoulder instability exacerbates the damage [32]. Furthermore, the mechanism of SLAP tears is similar to partial tears of the subscapularis and supraspinatus, including fall on an outstretched hand and an overhead throwing motion [41, 42].

### 7.6. Association with Anterior Dislocation of the Humeral Head

As stated above, anterior dislocation may be associated with subscapularis tendon tears, and such injuries coincide with humeral avulsion of the glenohumeral ligament (HAGL), specifically the inferior glenohumeral ligament [42]. Other findings associated with anterior dislocation include the Hill–Sachs deformities and the Bankart lesions (Figure 9) [40, 42, 43]. MRA has been reported to show a “J”-shaped inferior glenohumeral ligament with extravasation of contrast from the joint space [44].

## 8. Conclusion

Subscapularis tendon and neighboring anatomy pathology can be difficult to diagnose on physical examination and imaging. Failure to address tears to the subscapularis tendon can lead to delayed surgical repair, ongoing pain, and limited function and stability of the glenohumeral joint. Attention to the subscapularis tendon is rising, with a greater understanding aiding a systematic approach for better diagnosis. Increased perception for the subscapularis tendon, the biceps pulley, and rotator interval not only helps patients improve their quality of life but bring this pathology out of hiding.

## Data Availability

No data were used in this study.
